# Novel *One-Pot* Green Synthesis of Indolizines Biocatalysed by *Candida antarctica* Lipases

**DOI:** 10.3390/md11020431

**Published:** 2013-02-06

**Authors:** Rodica Mihaela Dinica, Bianca Furdui, Ioana Otilia Ghinea, Gabriela Bahrim, Simon Bonte, Martine Demeunynck

**Affiliations:** 1 Department of Chemistry, Physics and Environment, Faculty of Science and Environment, “Dunarea de Jos” University of Galati, 111 Domneasca Street, Galati 800201, Romania; E-Mail: rodinica@ugal.ro; 2 Department of Food Science, Food Engineering and Applied Biotechnology, Faculty of Food Science and Engineering, “Dunarea de Jos” University of Galati, 111 Domneasca Street, Galati 800201, Romania; E-Mails: ioana.ghinea@ugal.ro (I.O.G.); gabriela.bahrim@ugal.ro (G.B.); 3 Département Pharmacochimie Moléculaire, UMR 5063 & FR 2607, CNRS/Université de Grenoble, 38041 Grenoble cedex 9, France; E-Mail: simontlse.12@gmail.com

**Keywords:** indolizine, *Candida antarctica* lipase, *one-pot* reaction, cycloaddition, ylide

## Abstract

Marine microorganisms are of considerable interest as a promising source of enzymes with unsuspected potentials as catalysts for chemical synthesis. We describe here an efficient method for *one-pot* indolizine synthesis that has been developed using lipase A and lipase B from *Candida antarctica* as biocatalysts. As showed by HPLC/MS analysis, the yield in indolizines was higher in the presence of the biocatalyst than in absence of enzyme. Lipase A, from *Candida antarctica*, showed high catalytic activity and selectivity for the cycloaddition reactions. When the reactions were performed under ultrasound irradiation, the *Candida antarctica* lipase catalyzed reactions yielded pure indolozines, in good yields and in very short time.

## 1. Introduction

The indolizine ring is an important structural system frequently found in natural products and has been used as an important skeleton in pharmaceutics because of its interesting and promising biological properties [1,2,3]. Both natural and synthetic derivatives have been identified as anticancer, anti-tuberculosis, analgesic, and antioxidant agents. Several data point also to the potential of indolizine derivatives as antimicrobial agents [4,5], calcium entry blockers [6], antioxidants [7] or inhibitors of 15-lipooxygenase [8]. As a result, different approaches have been reported in the literature for their synthesis [3,9]. The cycloaddition of ylides with alkynes emerges as one of the most general, and widely used reactions in indolizine ring formation [10]. However, long reaction time, elevated thermal conditions, expensive and/or toxic metals catalysts, and environmentally hazardous organic solvents are generally required. 

We are interested in the synthesis of a variety of indolizine derivatives, and in the study of their biological, physical, and spectroscopic properties [11,12,13]. More recently we have initiated a project dealing with the use of microorganisms, or isolated enzymes, to design greener alternatives to known chemical processes.

Indeed, enzymes such as lipases are well-known both at the lab-bench and for industrial processes, and their use is widespread, especially when regio- or stereoselectivity is required. Microorganisms from marine sources have been, so far, little investigated in the search of new biocatalysts [14,15]. However, organisms originating from the Antarctic seawater are known to produce enzymes that are able to function at extremely low temperatures, and are therefore useful as novel tools in biotechnology, particularly for pharmaceuticals and the food industry. Antarctic yeast *Candida antarctica* is probably one of the most useful microorganism, which has been cited in numerous patents, and has also been used in a variety of commercial applications. The lipases A and B isolated from *Candida antarctica* (and their immobilized forms CAL A and CAL B) catalyze a variety of chemical reactions including ester hydrolysis, transesterification, and amide synthesis [16,17,18]. These enzymes are used advantageously in organic synthesis or biotechnology because of their low cost, good stability in organic solvents, absence of co-factors requirement, and large pH operating range. CAL A and CAL B are able to work with various types of substrates, and may be stereo-, chemo-, and regioselective. 

We report here an original and efficient method for the synthesis of substituted bis-indolizines via an enzyme catalyzed three-component reaction starting from 4,4′-bipyridine, α-bromo carbonyl reagents and activated alkynes. The reactions were carried out in an aqueous buffer solution in the presence of *Candida antarctica* enzymes (CAL A or CAL B). Our *one-pot* transformation involved the cycloaddition of a dipolarophile with a stable ylide, issued from the 4,4′-bipyridinium diquaternary salt, formed *in situ*. 

## 2. Results and Discussion

**Figure 1:**
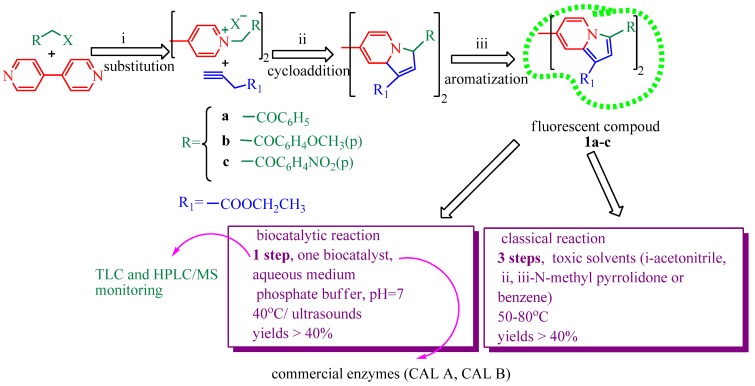
Synthesis of bisindolizines.

Several methods have been developed for the synthesis of indolizine derivatives, including recent one-pot multicomponent reactions [19,20]. In a previous paper, we reported the cycloaddition reaction of 4,4′-bipyridinium diquaternary salts with various alkynes that gave the corresponding fluorescent dyes in good yields [11,12]. The conditions are presented in Figure 1 as the classical reaction.

We designed the first example of a *one-pot* biocatalytic bis-indolizine ring formation and compared this process to the classical reaction. The reactions were realized starting, as previously reported, from 4,4′-bipyridine, halide derivatives (ω-bromacetophenones), and ethyl propiolate, in a pH 7 phosphate buffer solution, and stirred at 40 °C, either using a thermoshaker or under ultrasound activation (Figure 1). The *one-pot* reactions were carried out in the presence and in the absence of lipases to evidence the biocatalytic effect. Both classical and catalyzed reactions have given the same indolizine products **1a**–**c**.

Indeed, the choice of the reaction parameters (nature of the solvent, temperature, time, and substrate concentration) is an important step in biocatalysis. Water is the solvent of choice as the enzyme-bound water is essential for catalysis and serves as a lubricant for the enzyme. The temperature can be used to influence the viscosity of the medium, which in turn influences the solubility of substrates and products, enzyme activity, and even the biocatalyst denaturation. The molar ratio of reagents also modulates the product yield. Thus, for the *in situ* formation of the 4,4′-bipyridinium diquaternary salts from 4,4′-bipyridine and ω-bromacetophenones, and for the subsequent cycloaddition, excesses of alkylating agent (ω-bromacetophenones) and dipolarophile (ethyl propiolate) were used. The selected reaction conditions for the cycloaddition using *Candida antarctica* enzymes were using water as the solvent and excess (three equivalents) of ω-bromacetophenones and ethyl propiolate. 

**Figure 2:**
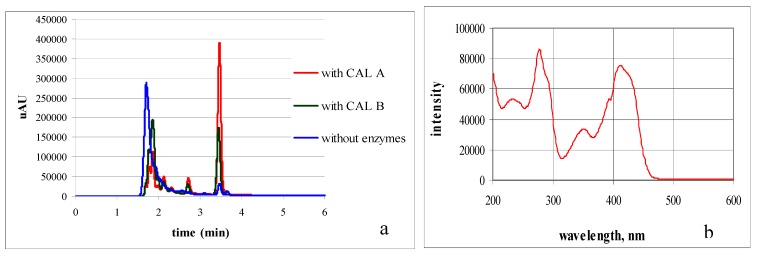
Hplc chromatograms of the *one-pot* reaction mixtures after 48 h of shaking in a thermoshaker (**a**) and UV-VIS spectra (**b**) corresponding to the compound eluting at 3.4 min and corresponding to the indolizine **1a**.

The *one-pot* transformations were monitored by high performance liquid chromatography (HPLC) using a system equipped with a diode array detector. Figure 2a displays the chromatogram profiles of the reactions of the 4,4′-bipyridine with ω-bromacetophenone and ethyl propiolate, with and without enzymes, after 48 h of stirring at 40 °C in a thermoshaker. The chromatograms showed two important peaks: a peak eluting at 1.8 min, probably corresponding to the intermediary diquaternary salt, and a peak at 3.4 min, identified as the final indolizine **1a**. This last peak showed, in its corresponding UV-Vis spectra (Figure 2b), several intense absorption bands (231 nm, 277 nm, 352 nm, 411 nm) that are characteristics of the indolizine ring. This observation confirmed that the cycloaddition reaction occurred. 

**Figure 3:**
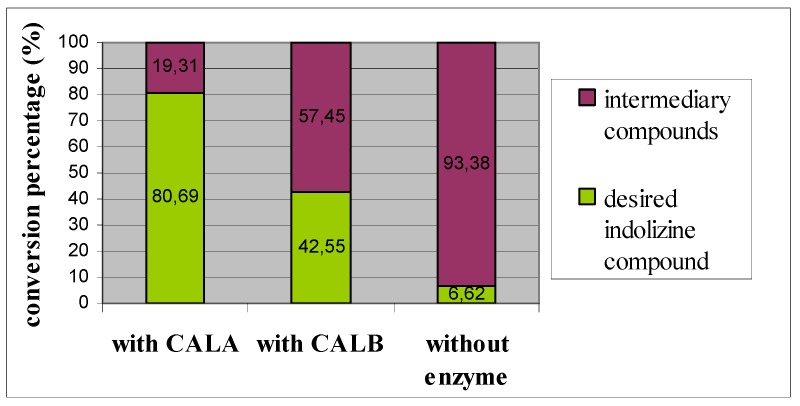
Conversion percentage for enzymatic and non-enzymatic *one-pot* synthesis of indolizine **1a**.

The chromatograms also showed that the concentration in indolizinic compound was greater in the presence of the biocatalysts. The conversion ratio in indolizine **1a**, calculated from HPLC chromatograms, is illustrated in Figure 3. 

After 48 h of reaction in phosphate buffer solution at 40 °C, it was observed that starting materials were converted to indolizine compounds in all samples, but better yields of indolizines **1a**–**c **were obtained when CAL A and CAL B were used as biocatalysts. Comparing the two enzymes, CAL A showed better activity for the cycloaddition reaction with higher yields in the desired products, **1a**–**c **may be due to the lower thermostability and higher acidity of CAL B compared with CAL A [21]. The good yield observed in the presence of the *Candida antarctica* enzymes, and the poor results obtained in the absence of enzymes, were attributed to the ability of the enzymes to perform the cycloaddition reactions under these buffer conditions. 

**Figure 4:**
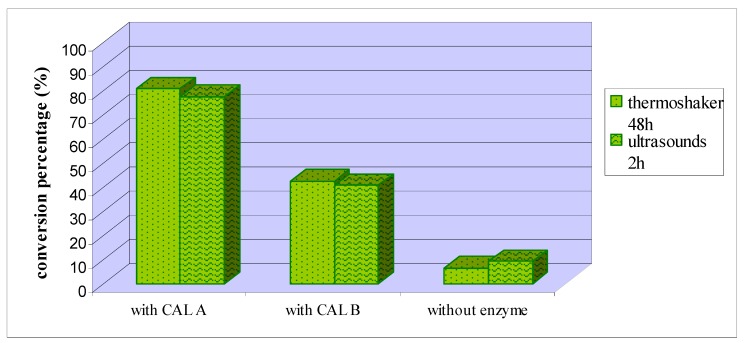
Conversion percentage to indolizine **1a** in enzymatic and non-enzymatic *one-pot* synthesis, carried out with and without ultrasounds.

The enzymatic and non-catalyzed *one-pot* syntheses were then carried out with stirring and heating at 40 °C in an ultrasound bath, for two hours. As shown in Figure 4, ultrasounds activated the reactions, as similar conversion rates were obtained but with reaction times significantly reduced (from 48 h to 2 h).

These conditions were then applied to the lipase catalyzed synthesis of indolizines **1b** and **1c**, with similar results as for indolizine **1a**. In the HPLC analysis, indolizines **1b** and **1c** appeared at 6.45 min and at 6.03 min respectively.

As a conclusion, this biocatalytic method used inexpensive and readily available solvents (water), and allowed *N*-alkylation of 4,4′-bipyridine and subsequent cycloaddition reaction to occur in *one-pot*, thus limiting the number of experimental steps. The reactions took place in a more environmentally friendly way than the classical two-step reactions. It is worth mentioning that both classical and enzyme catalyzed reactions gave the same indolizine products **1a**–**c**, thus indicating that the ester group present in **1a**–**c** (R=COOEt) is not a substrate for the studied lipases in these conditions. Indeed, in a separate experiment, we checked that the ester group present in indolizine **1a** was not hydrolysed in the presence of CAL A.

This procedure represents an eco-friendly regioselective approach to the indolizine core formation.

Our synthetic approach, which involves the cycloaddition of *in situ* generated ylides, in aqueous medium, turned out to be the first example of a *one-pot* bio-preparation for indolizines.

## 3. Experimental Section

### 3.1. Enzymes and Chemicals

The lipases used were immobilized lipases from *Candida antarctica*, CAL A (Novozyme 735, 1.6 U/mg) and CAL B (Novozyme 435, ≥10000 U/g). All the other reagents used for synthesis were purchased from Aldrich, Fluka, and Merck companies. The solvents used in high performance liquid chromatography are of analytic grade from Merck^®^. A Milli-Q System^®^ (Bedford, MA, USA) was used to purify the water. 

### 3.2. Equipment and Chemical Analysis

The biocatalyzed reactions were performed in an orbital flasks thermoshaker (Biosan) or in an ultrasound bath (Bandelin Sonrex Digitech). The progress of each reaction was monitored using an HPLC Thermo Scientific system, using a C18 BDS column and acetonitrile as mobile phase, with a flow rate of 1 mL/min and injection volume of 10 μL. Melting points were recorded with a Büchi Melting Point B-540. ^1^H NMR and ^13^C NMR spectra were recorded with a Bruker 400 Ultrashield (400 MHz) spectrometer operating at room temperature. Deuterated CDCl_3_ was used as solvent. Abbreviations for data quoted are: s, singlet; d, doublet; t, triplet; q, quartet; dd, doublet of doublets; m, multiplet. IR spectra were recorded from 4000 to 650 cm^−1^ with a Perkin-Elmer Spectrum 100 instrument by total reflectance on a CdSe crystal. Elemental analyses (C, H, N) were performed with a Fisons Instruments 1108 CHNS–O elemental analyzer. The atmosphere pressure chemical ionization (APCI) mass spectra were measured on Thermo Scientific HPLC/MSQ Plus. 

### 3.3. Procedure for One-Pot Non-Enzymatic Reactions

The 4,4′-bipyridine (1.2 mmol), halide derivatives (3.6 mmol), and ethyl propiolate (3.6 mmol), were added to Erlenmeyer flasks (50 mL) containing phosphate buffer solutions (250 mM, pH 7, 12 mL). The mixtures were stirred in a rotary shaker (200 rpm) at 40 °C for 48 h or in ultrasonic bath for 2 h.

### 3.4. Procedure for One-Pot Enzymatic Reaction

To a 50 mL Erlenmeyer flask containing phosphate buffer solutions (250 mM, pH 7, 12 mL) and enzyme (CAL A or CAL B) (10 mg) was added the 4,4′-bipyridine (1.2 mmol), halide derivatives (3.6 mmol), and ethyl propiolate (3.6 mmol). The reaction mixture was stirred in a rotary shaker (200 rpm) at 40 °C until maximum conversion of the starting material (48 h) or under ultrasounds (2 h). 

### 3.5. Procedure for Extraction of Indolizines

All reactions were monitored by HPLC and, after the appropriate conversion ratio, the mixture was filtered and the aqueous phase was extracted using chloroform. The yellow organic phase was dried over MgSO_4_ and then filtered. The solvent was evaporated under reduced pressure and the residue was precipitated with methanol giving the bis-indolizine products in 67% (**1a**), 60% (**1b**), 66% (**1c**) yields for CAL A and 28% (**1a**), 25% (**1b**), 27% (**1c**) yields for CAL B.

### 3.6. Analysis of the Final Indolizine Products **1a–c**

The products of biocatalyzed reactions were analyzed by IR, NMR, MS, elemental analysis and the obtained data were identical with those of the substituted 7,7′-bis-indolizines previously obtained and published by us [11]. 

**1,1′-diethyldicarboxylate-3,3′-benzoyl-7,7′-bis(indolizine) (1a)**: yellow crystals, mp 273–275 °C; IR (ATR, cm^−1^): 3139 (l, CH_arom_); 2979 (l, CH_alif_); 1700 (s, C=O_ester_); 1645 (s, C=O); 1524, 1512, 1479, 1459, 1427 (s, C=N, C=C_arom_); 1341 (s, CO_ester_) 1216, 1167 (s, C–O–C); 1071 (s, C–N); ^1^H-NMR (400 MHz, CDCl_3_, TMS) δ/ppm: 9.98 (d, *J* = 7.5, 2H); 8.77(d, *J* = 1.2, 2H); 7.78-7.88 [m, 6H: 4H, 2H (7.82)]; 7.42–7.73 [m, 8H: 4H, 2H, 2H (7.52, *J* = 7.5, *J* = 1.2)]; 4,4 (q, *J* = 7.2, 4H); 1,43 (t, *J* = 7.2, 6H); ^13^C-NMR (400 MHz, CDCl_3_, TMS δ/ppm): 185.67 (2C=O), 163.97 (2C=O_ester_), 139.78 (2C), 139.70 (2C), 136.61 (2C), 131.74 (2CH), 129.53 (2CH), 129.33 (2CH), 129.05 (4CH), 128.52 (4CH), 122.90 (2C), 116.96 (2C), 113.53 (2CH), 107.55 (2C), 60.37 (2CH_2_), 14.59 (2CH_3_); MS (APCI+): *m/z*: 585 [M + H]^+^; anal. C 73.72; H 5.14; N 4.76%, calcd. for C_36_H_28_N_2_O_6_, C 73.96, H 4.83, N 4.79%. 

**1,1′-diethyldicarboxylate-3,3′-bis(p-methoxy-benzoyl)-7,7′-bis(indolizine) (1b)**: yellow crystals, mp 290–291 °C; IR (ATR, cm^−1^): 3140 (l, CH_arom_); 2977 (l, CH_alif_); 1700 (s, C=O_ester_); 1665 (s, C=O); 1597, 1508, 1463 (s, C=N, C=C_arom_); 1339 (s, CO_ester_) 1249, 1220, 1167 (s, C–O–C); 1077 (s, C–N); ^1^H-NMR (400 MHz, CDCl_3_, TMS) δ/ppm: 9.98 (d, *J* = 7.5, 2H); 8.80(d, *J* = 1.2, 2H); 7.79 (d, *J* = 7.5, 2H); 7.76 (s, 2H); 7.33 (d, *J* = 8.8, 4H); 7.03 (d, *J* = 8.8, 4H); 4.43 (q, *J* = 7.2, 4H); 3,93 (s, 6H) 1,43 (t, *J* = 7.2, 6H); ^13^C-NMR (400 MHz, CDCl_3_, TMS δ/ppm ): 186.06 (2C=O), 165.28 (2C=O_ester_), 163.71 (2C), 138.57 (2C), 136.57 (4CH), 132.42 (2C), 131.81 (2CH), 129.04 (2CH), 122.2 (4CH), 122 (2CH), 117.72 (2CH), 114 (4CH), 113.98 (2CH), 107 (2C), 61.09 (CH_2_), 56,03 (OCH_3_), 14,73 (CH_3_); MS (APCI+): *m/z*: 645 [M + H]^+^; anal. C 70.92; H 4.88; N 4.28%, calcd. for C_38_H_32_N_2_O_8,_ C 70.80, H 5.00, N 4.35%.

**1,1′-diethyldicarboxylate-3,3′-bis(p-nitro-benzoyl)-7,7′-bis(indolizine) (1c)**: yellow crystals, mp 271–272 °C; IR (ATR, cm^−1^): 3359 (l, CH_arom_); 2978 (l, CH_alif_); 1700 (s, C=O_ester_); 1656 (s, C=O); 1464, 1428 (s, C=N, C=C_arom_); 1521, 1338 (s, NO_2_); 1208, 1169 (s, C–O–C); 1077 (s, C–N); ^1^H-NMR (400 MHz, CDCl_3_, TMS) δ/ppm: 10.13 (d, *J* = 7.5, 2H); 8.99(d, *J* = 1.2, 2H); 8.03 (d, *J* = 8.8, 4H); 7.8 (d, *J* = 8.8, 4H); 7.48 (d, *J* = 7.5, 2H ); 7.42 (s, 2H); 4.4 (q, *J* = 7.2, 4H); 1.43 (t, *J* = 7.2, 6H); ^13^C-NMR (400 MHz, CDCl_3_, TMS δ/ppm ): 184.15 (2C=O), 164.52 (2C=O_ester_), 150.16 (2C), 143.06 (2C); 138.14 (2C); 132.60 (2C); 129.86 (4CH); 129.14 (2CH); 127.46 (2CH); 123.52 (4CH); 122.74 (2C); 118.28 (2CH); 113.07 (2CH); 108.16 (2Cquat); 60.53 (2CH_2_); 14.29 (2CH_3_); MS (APCI+): *m/z*: 675 [M + H]^+^; anal. C 64.15; H 4.11; N 8.26%, calcd. for C_36_H_26_N_4_O_10_, C 64.09, H 3.88, N 8.31%. 

## 4. Conclusions

Our synthetic approach is the first example for biocatalyzed *one-pot* synthesis for indolizines, which involves cycloadditions of ethyl propiolate with the *in situ* generated ylides, in aqueous media.

The best results were obtained when the *Candida antarctica* lipase catalyzed the biotransformation of 4,4′-bipyridine, halide derivatives, and ethyl propiolate to form indolizine compounds. A better yield of indolizine was obtained when CAL A was used as a biocatalyst. The catalytic reactions with lipase from *Candida antarctica* produced almost pure indolizines with good yields and in very short time under ultrasound activation. As mentioned above, the lipase catalyzed synthesis was performed in water, a green solvent compared to the organic solvents usually required for the classical methodologies.

The increasing importance of safety, health, and environmental issues as well as initiatives in green and sustainable chemistry, white and industrial biotechnology, and the new perspectives of molecular economy plainly justify the work done to develop this biocatalytic approach using enzymes for indolizine synthesis. Further developments of this methodology are under investigations. In particular, the process will be extended to the synthesis of variously substituted mono- and *bis*-indolizines of biological interest.
